# Degradation of band3 and PRDX2 in erythrocytes during severe acute GVHD

**DOI:** 10.1002/jha2.660

**Published:** 2023-02-15

**Authors:** Masayuki Nagasawa

**Affiliations:** ^1^ Department of Pediatrics Musashino Red Cross Hospital Musashino Tokyo Japan; ^2^ Department of Pediatrics and Developmental Biology Tokyo Medical and Dental University Tokyo Japan

**Keywords:** band3, calpain‐1, erythrocytes, GVHD, oxidative stress, peroxiredoxin 2

## Abstract

We investigated the proteins of erythrocytes from stem cell transplantation patients and found decreased expression of band3 and C‐terminal‐truncated peroxiredoxin 2 (PRDX2) only during severe graft‐versus‐host disease (GVHD), using time‐of‐flight mass spectrometry (TOF‐MS) analysis and Western blotting. During the same period, PRDX2 dimerization and calpain‐1 activation were observed, indicating severe oxidative stress. We also found a putative cleavage site for calpain‐1 in the C‐terminal‐truncated site of PRDX2. Decreased band3 expression impairs the plasticity and stability of erythrocytes, and C‐terminal‐truncated PRDX2 induces irreversible dysfunction of antioxidant activity. These effects may exacerbate microcirculation disorders and the progression of organ dysfunction.

**Abbreviations**
GVHD: graft‐versus‐host disease
PRDX: peroxiredoxin
ROS: reactive oxygen species
SCT: stem cell transplantation
TOF‐MS: time‐of‐flight mass spectrometry

## 1 INTRODUCTION

Reactive oxygen species (ROS) are not only produced in a basic electron transport system of the life activity, but also from various external stimulations and inflammation. Each organism has various mechanisms to remove ROS because they are harmful to the living body. However, excessive production of ROS beyond the permissible level causes serious damage to the living body. It has been reported that ROS are involved and play crucial roles during the progression of diabetes and arteriosclerotic diseases.

Erythrocytes are the most powerful buffer against ROS‐induced in vivo oxidative stress. Erythrocytes carry oxygen from the lungs to the entire body and are consequently exposed to oxidative stress. The most abundant and powerful substance against oxidative stress in erythrocytes is peroxiredoxin 2 (PRDX2), which removes ROS via the glutathione system [1]. PRDX2 has two cysteine residues, and the thiol‐dependent hydrogen peroxide removal activity is regulated by the thioredoxin/NADPH reduction mechanism. PRDX2 is located in the cytoplasm; it forms a dimer when oxidized in the presence of reactive oxygen and is then translocated to bind to the cell membrane [2, 3]. Reportedly, PRDX2‐null mice present hemolytic anemia [4].

During hematopoietic stem cell transplantation (SCT), ROS are induced by acute and chronic graft‐versus‐host disease (GVHD) as well as anticancer drugs and irradiation used as a conditioning regimen. In a mouse model of acute GVHD, erythrocytes are subjected to oxidative stress [5].

## 2 RESULTS AND DISCUSSION

**FIGURE 1:**
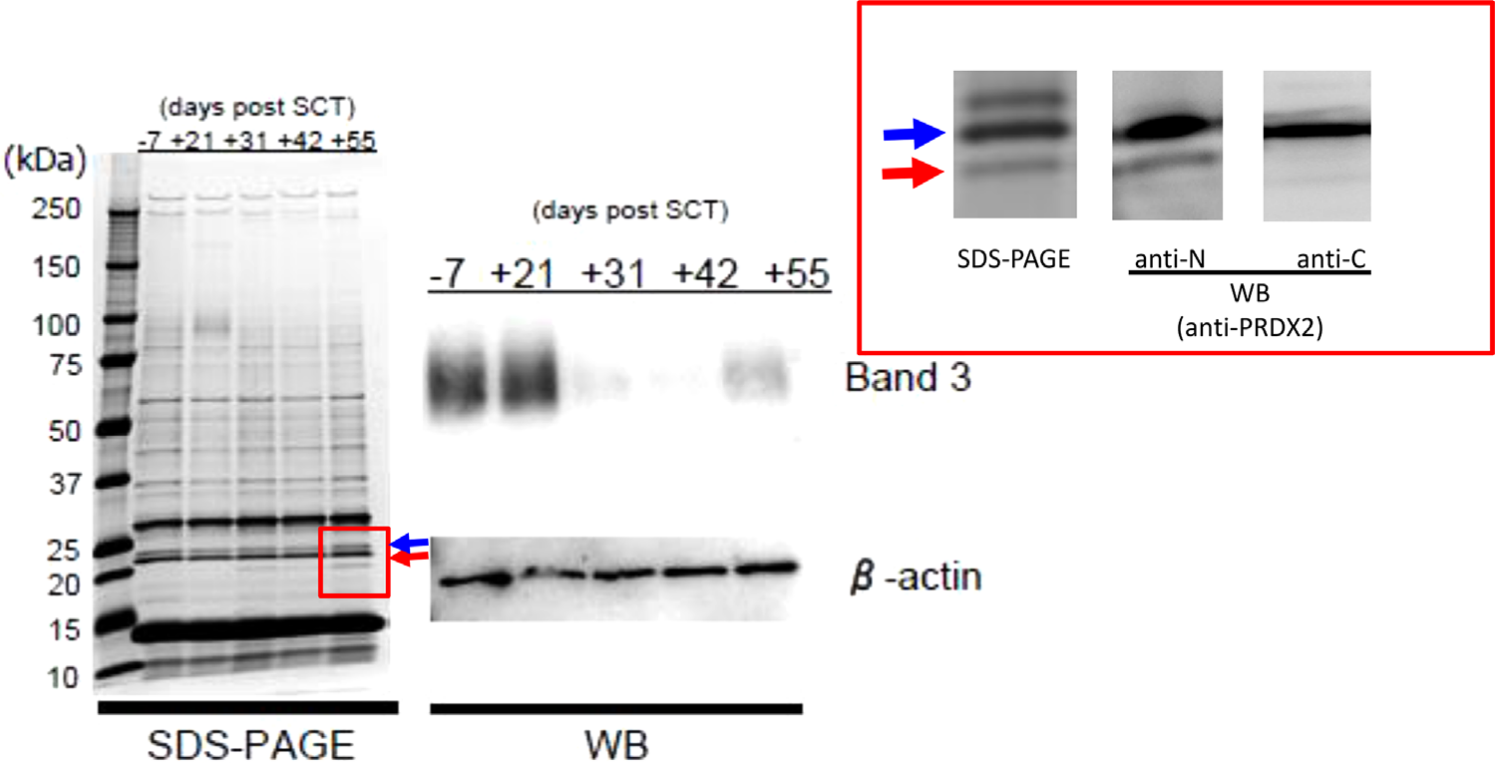
Analysis of erythrocyte proteins by SDS‐PAGE and Western blotting. Erythrocytes from the stem cell transplantation (SCT) patients were sequentially obtained as indicated. Expression of band3 was reduced between day 31st and 55th after SCT, when severe graft‐versus‐host disease (GVHD) developed (left). During the same period, a new band of 20 kDa was detected (encircled by a red box), which was determined to be C‐terminus‐truncated peroxiredoxin 2 (PRDX2) by Western blotting (right).

We investigated the proteins of erythrocytes from SCT patients sequentially using SDS‐PAGE and found the decreased expression of band3 during severe GVHD, which was determined by Western blotting (Figure 1). Band3 is the most abundant membrane protein in erythrocytes that performs two functions: electroneutral chloride and bicarbonate exchange across the plasma membrane and maintaining the plasticity and stability of erythrocytes, enabling stable microcirculation [6]. Furthermore, a new band of approximately 20 kDa was detected along with decreased expression of band3. The 20 kDa band was proved to be C‐terminus‐truncated PRDX2 by time‐of‐flight mass spectrometry (TOF‐MS) analysis. This result was confirmed by Western blot analysis using two antibodies that specifically recognized the N‐terminus and C‐terminus of PRDX2, respectively (Figure 1). The cleavage site was speculated to be located between the 155th and 198th amino acid residue from TOF‐MS analysis. SDS‐PAGE analysis under non‐reduction conditions revealed that PRDX2 was dimerized when a decrease in band3 expression and a 20 kDa band were observed (Figure S1), which indicated that erythrocytes were exposed to severe oxidative stress.

Examination of the erythrocyte membrane proteins of 16 recipients of allogeneic hematopoietic cells showed that these changes were detected only in six cases with GVHD3 or 4, but not in cases without GVHD or with GVHD1 or 2 (Table S1). Long‐term analysis showed that reduced expression of band3 recovered and truncated PRDX2 disappeared after the resolution of GVHD (Figure S2).

**FIGURE 2:**
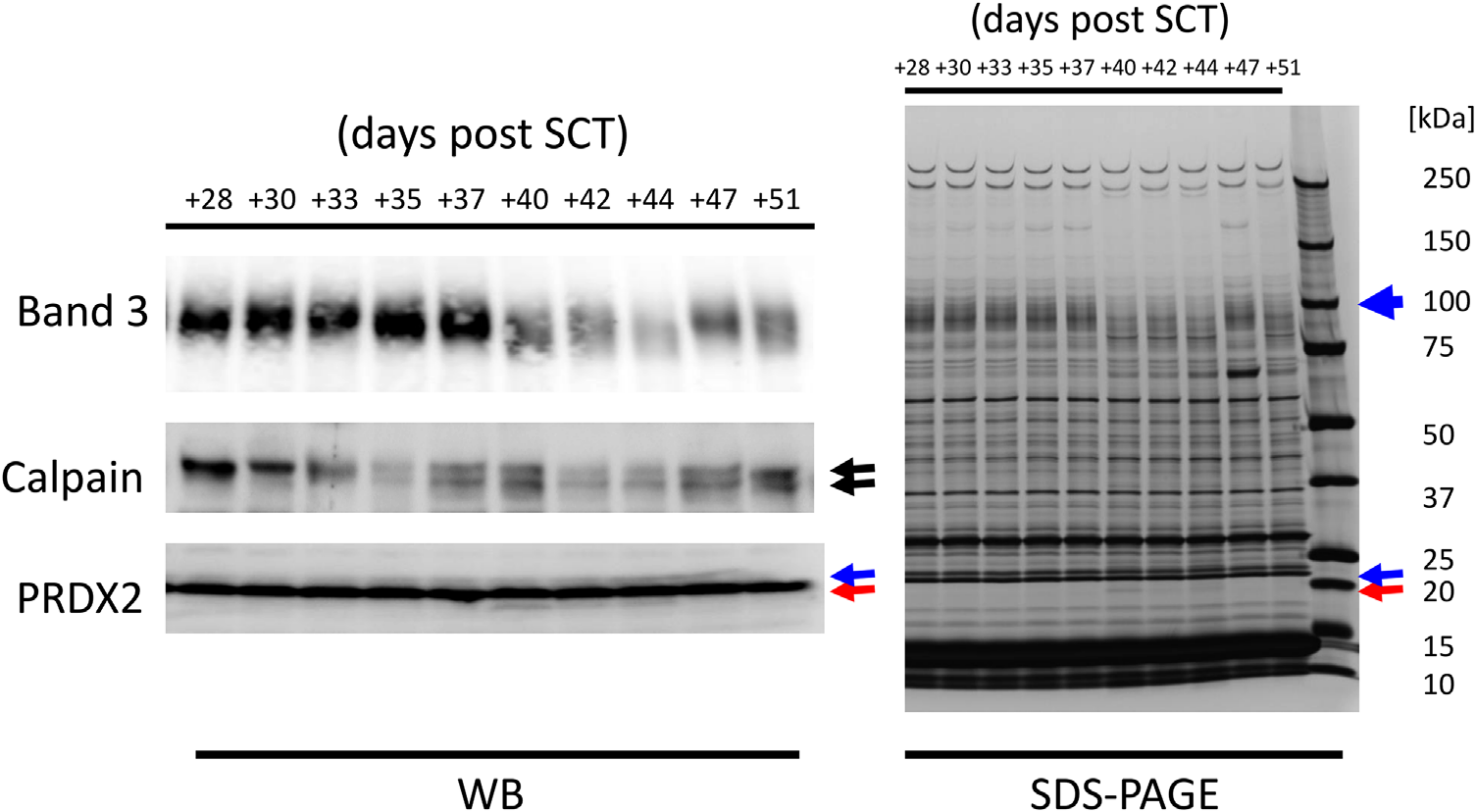
Calpain was activated during severe graft‐versus‐host disease (GVHD). Erythrocyte proteins obtained as indicated were analyzed by SDS‐PAGE and Western blotting. Activation of calpain was detected, along with decreased expression of band3 and the appearance of truncated peroxiredoxin 2 (PRDX2).

However, it has been reported that the erythrocyte protease calpain is activated through autoproteolysis induced by oxidative stress via calcium, and moves to the cell membrane from the cytoplasm [7]. Additionally, anoxia has been reported to activate calpain [8]. Furthermore, activated calpain degrades band3 [9], and the analysis of calpain‐1‐knockout mice has revealed that activated calpain degrades band3 in vivo [10]. According to these reports, we examined calpain autoproteolysis in erythrocytes and found that calpain activation was detected in accordance with the decreased band3 expression and 20 kDa band appearance (Figure 2). PRDX2 consists of 198 amino acids and recognizes several putative cleavage sites for calpain [11], out of which the 157th and 182nd amino acid residue sites are determined at the C‐terminus of PRDX2.

It might be possible that degradation of band3 or PRDX2 is induced by physical impact, such as hemolysis or fragmentation in thrombotic microangiopathy. However, it is difficult to explain the constant cutting site of PRDX2 in this context. Furthermore, none of the patients we examined presented clinically determined thrombotic microangiopathy or hemolytic disorder before the onset of degradation of band3 and PRDX2.

The decreased expression of band3 affects the plasticity and stability of erythrocytes [12], and the C‐terminal‐truncated PRDX2 presents irreversible dysfunction [13, 14], resulting in defective antioxidant activity of erythrocytes. These phenomena may exacerbate microcirculation disorders, and further induce the progression of organ dysfunction in severe GVHD. Calpain inhibitors have been reported to improve the plasticity of erythrocytes in an in vivo mouse model [15]. In light of our observations described above, it is speculated that calpain activation in severe GVHD is involved in progressive organ dysfunction and deterioration through impaired microcirculation induced by the decreased antioxidant activity and plasticity of erythrocytes and that calpain may become a target for therapeutic strategies to minimize organ damage in severe GVHD.

## AUTHOR CONTRIBUTIONS

Masayuki Nagasawa conceptualized and designed the study, analyzed the data, and wrote the manuscript.

## CONFLICT OF INTEREST STATEMENT

The author declares no conflicts of interest.

## ETHICS STATEMENT

This study was performed in compliance with the ethical treatment policy of human and animal research participants and the Declaration of Helsinki. This study was approved by institutional review board of Tokyo Medical and Dental University.

## PATIENT CONSENT STATEMENT

Written informed consent was obtained from the guardians of each SCT patient.

## Supporting information

Supporting Information

## Data Availability

Data are available on request from the author.
