# Supplementary material for: Degradation of band3 and PRDX2 in erythrocytes during severe acute GVHD
Source: EJHaem. 2023 Feb 15;4(2):459–62. doi: 10.1002/jha2.660 (PMC10188505; doi:10.1002/jha2.660)
Supplement: Supplementary file 1 — Supporting Information [file JHA2-4-459-s001.pptx]

## Slide 1
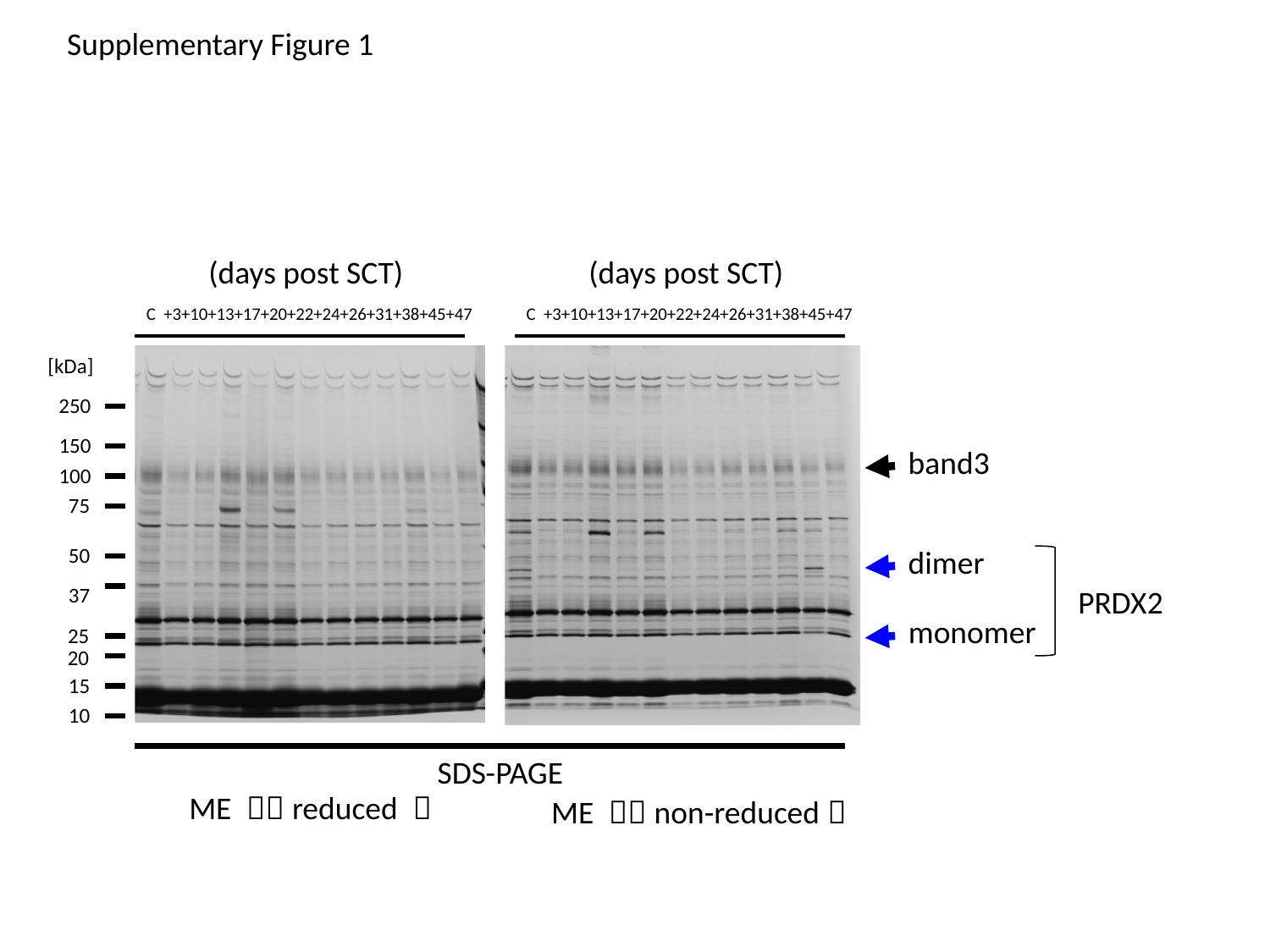

Supplementary Figure 1
(days post SCT)
(days post SCT)
C +3+10+13+17+20+22+24+26+31+38+45+47
C +3+10+13+17+20+22+24+26+31+38+45+47
[kDa]
250
150
band3
100
75
50
dimer
37
PRDX2
monomer
25
20
15
10
SDS-PAGE
ME ＋（reduced ）
ME －（non-reduced）

## Slide 2
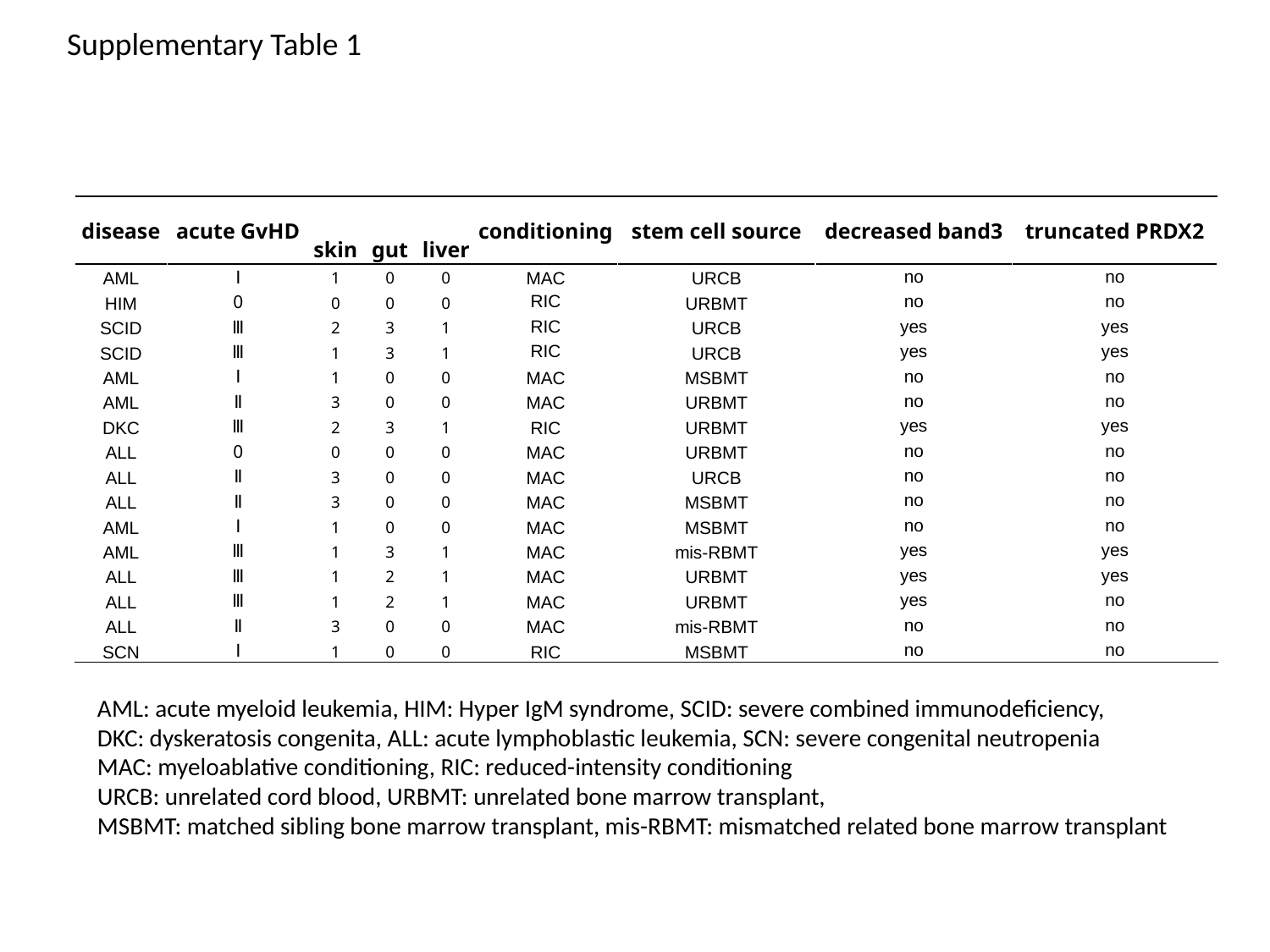

Supplementary Table 1
| disease | acute GvHD | | | | conditioning | stem cell source | decreased band3 | truncated PRDX2 |
| --- | --- | --- | --- | --- | --- | --- | --- | --- |
| | | skin | gut | liver | | | | |
| AML | Ⅰ | 1 | 0 | 0 | MAC | URCB | no | no |
| HIM | 0 | 0 | 0 | 0 | RIC | URBMT | no | no |
| SCID | Ⅲ | 2 | 3 | 1 | RIC | URCB | yes | yes |
| SCID | Ⅲ | 1 | 3 | 1 | RIC | URCB | yes | yes |
| AML | Ⅰ | 1 | 0 | 0 | MAC | MSBMT | no | no |
| AML | Ⅱ | 3 | 0 | 0 | MAC | URBMT | no | no |
| DKC | Ⅲ | 2 | 3 | 1 | RIC | URBMT | yes | yes |
| ALL | 0 | 0 | 0 | 0 | MAC | URBMT | no | no |
| ALL | Ⅱ | 3 | 0 | 0 | MAC | URCB | no | no |
| ALL | Ⅱ | 3 | 0 | 0 | MAC | MSBMT | no | no |
| AML | Ⅰ | 1 | 0 | 0 | MAC | MSBMT | no | no |
| AML | Ⅲ | 1 | 3 | 1 | MAC | mis-RBMT | yes | yes |
| ALL | Ⅲ | 1 | 2 | 1 | MAC | URBMT | yes | yes |
| ALL | Ⅲ | 1 | 2 | 1 | MAC | URBMT | yes | no |
| ALL | Ⅱ | 3 | 0 | 0 | MAC | mis-RBMT | no | no |
| SCN | Ⅰ | 1 | 0 | 0 | RIC | MSBMT | no | no |
AML: acute myeloid leukemia, HIM: Hyper IgM syndrome, SCID: severe combined immunodeficiency,
DKC: dyskeratosis congenita, ALL: acute lymphoblastic leukemia, SCN: severe congenital neutropenia
MAC: myeloablative conditioning, RIC: reduced-intensity conditioning
URCB: unrelated cord blood, URBMT: unrelated bone marrow transplant,
MSBMT: matched sibling bone marrow transplant, mis-RBMT: mismatched related bone marrow transplant

## Slide 3
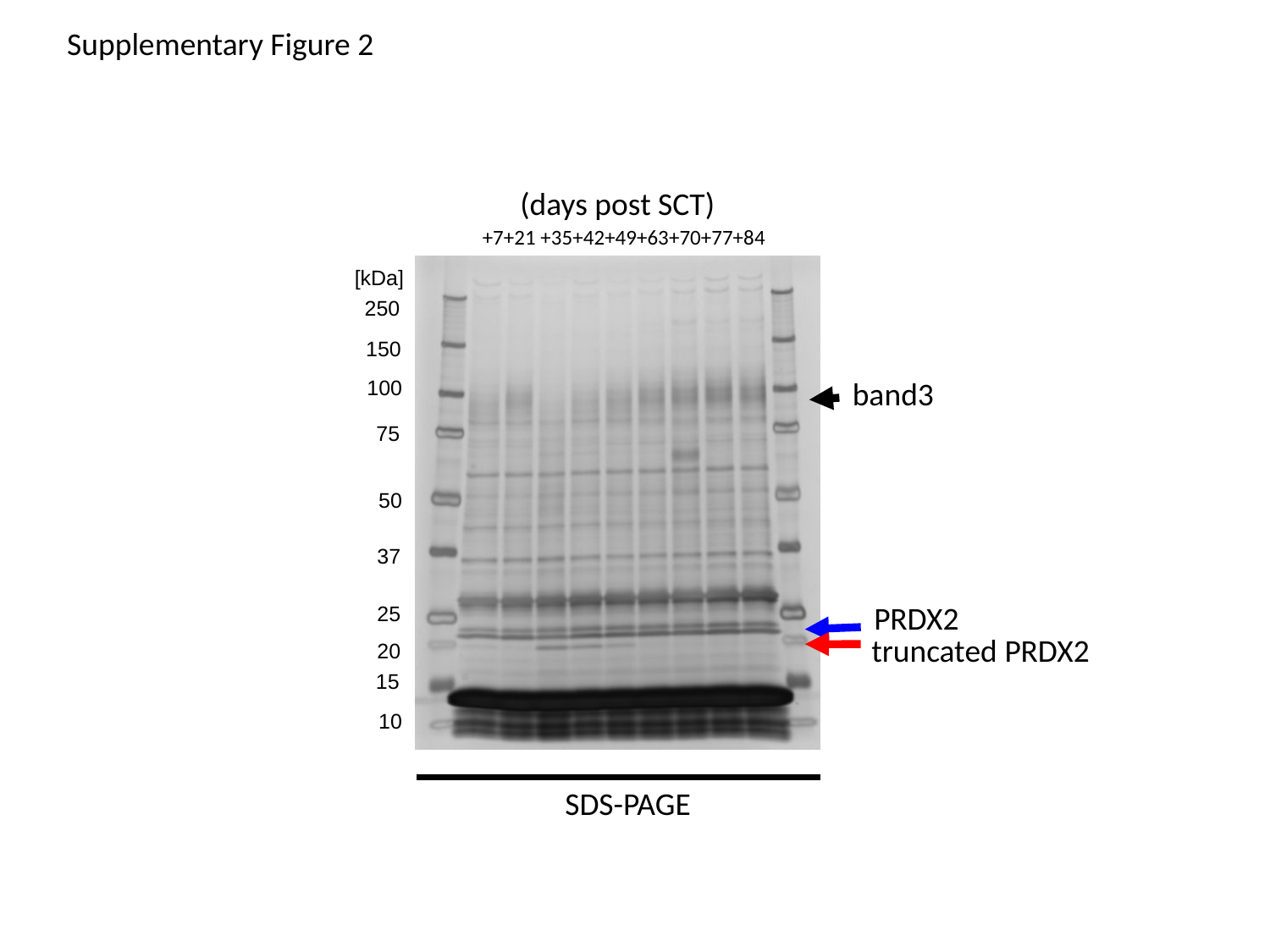

Supplementary Figure 2
(days post SCT)
 +7+21 +35+42+49+63+70+77+84
[kDa]
250
150
100
band3
75
50
37
PRDX2
25
truncated PRDX2
20
15
10
SDS-PAGE
